# Exploring the Acceptance of Entomophagy: A Survey of Italian Consumers

**DOI:** 10.3390/insects12020123

**Published:** 2021-01-29

**Authors:** Roberta Moruzzo, Simone Mancini, Fabio Boncinelli, Francesco Riccioli

**Affiliations:** 1 Department of Veterinary Sciences, University of Pisa, Viale delle Piagge 2, 56124 Pisa, Italy; roberta.moruzzo@unipi.it (R.M.); francesco.riccioli@unipi.it (F.R.); 2 Department of Agriculture, Food, Environment and Forestry, University of Florence, Piazzale delle Cascine 18, 50144 Firenze, Italy; fabio.boncinelli@unifi.it

**Keywords:** insect phobia, neophobia scale, logit model, insects

## Abstract

**Simple Summary:**

Population growth and the environmental impacts of food production have led several international agencies to focus on future prospects for more environmentally sound products. Edible insects could be one of those solutions. Entomophagy, the practice of eating insects by humans, is widespread in certain countries, while, historically, it has been avoided in others, such as Western countries. In this paper, we focus on Italian consumers and their acceptance of this novel food. The results highlight a certain phobia in relation to this new practice that could be kept under control by appropriate commercialization strategies.

**Abstract:**

Insect-based food is not common in Europe, because most people do not consider insects to be edible, but rather a threat and a health risk. Fear and refusal to eat a new food product introduced into a culture is called food neophobia, which results in a hesitation to trying and experimenting with new foods. Although there is significant interest in this novel sector, there is a lack of research on the link between rejection, the level of food neophobia, and consumer behavior related to the introduction of insects into the diet. In this study, through 420 questionnaires, a specific experimental scale of insects was introduced which, together with a neophobia scale, analyzed the probability and the intention of respondents to consume insects. Another issue tested has been their intention to eat food containing insects. We observed that the analyses of the two scales produced different results, confirming the need for a specific scale to measure “insect phobia”. This is important, since knowledge about consumer preferences for and barriers to using insects as human food sources is limited but necessary in order to set up commercialization strategies. The development of insect-based food offers physical health benefits and also improves the sustainability of the food industry.

## 1. Introduction

Interest in entomophagy (human consumption of insects) has been increasing [1] over the last few years. Many authors [2,3,4,5,6,7] have asserted that eating insects offers physical health benefits, and also improves sustainability and food security.

In 2013, the Food and Agricultural Organization (FAO) published the report “Edible Insects: Future Prospects for Food and Feed Security” [8], which analyzed the practice of collecting insects as a source of food and income, and examined its relative ecological impact on forest habitats. To date, there have been 2111 edible insect species recorded [9] and approximately 2 billion people, in over 130 countries, that use insects as food [8,10]. Toti et al. [11] noted that “insects are currently consumed as part of the daily diet in many developing and non-developing countries, like Africa, Asia, Latin America, and Oceania”. In recent years, in Europe, there has been encouragement for the entomophagy sector. Indeed, the European Union, through Regulation (EU) 2015/2283 on novel foods, regulated insect consumption for the first time in Europe [12]. Right during the writing of this article, the European Food Safety Authority (EFSA) published the first scientific opinion about insects as novel food [13]. The EFSA Panel affirmed that dried yellow mealworm (*Tenebrio molitor* larva) could be considered as a safe novel food following the Regulation (EU) 2015/2283. Considering this scientific opinion, a milestone in the EU insect sector, there is considerable potential in the next years that several other insect species could be used as food and feed in the EU.

Although an increased number of studies have analyzed the acceptance of entomophagy [14,15,16,17,18], Western societies continue to have attitudinal barriers to considering insects as food [1,19,20]. Particularly, in countries that have no recent history of eating insects, it has been difficult to accept the practice of entomophagy [21]. Piha et al. [22] argued that “as far as Europe is concerned, consumers in Northern countries are generally more inclined to consider edible insects as food than people in Central European countries”.

The literature on consumer acceptance of entomophagy is debatable. Some studies have identified an interest in entomophagy, particularly among young consumers [23,24,25]. On the other hand, other studies that stress the health and environmental benefits of entomophagy have shown that these benefits are not sufficient to motivate human consumption [19,26]. Finally, some studies have analyzed the practice of incorporating insects into food (i.e., insect flour) and have concluded that it is not enough for people to accept insects as food [27]. Thus, in recent years, some studies have tried to investigate the main factors affecting Westerners’ acceptance of entomophagy (see [28] for more details). One of the main factors cited is neophobia.

As described by Pliner et al. [29], “food neophobia is a psychological attitude, which refers to the individual’s unwillingness to try and the tendency to avoid novel food”, which reduces the probability of introducing insects into the diet [1,20,30,31,32,33,34,35].

Moreover, the attitudinal measure obtained from food neophobia needs to be correlated with a series of factors underlying the acceptance/rejection of novel foods (both personal and social factors), as codified by Mancini et al. [16]. Among the personal factors, disgust has been identified by several studies as a core barrier to eating insects [36,37,38]. Considering that Westerners view insects to be a pathogenic risk, foods containing insects are considered to be disgusting [38,39,40].

Other studies have focused on psychological barriers such as lack of familiarity [31,32,37,41,42], their appearance [43], previous beliefs concerning the appropriateness of insect consumption [42], and product preparation and presentation [27]. Additionally, insects are rarely eaten because they are not considered to be edible [15,32,39]; in particular, they are often viewed as pests and a risk to health [32,44] or associated with a sense of filth and danger [39].

It is thought that food neophobia and the abovementioned factors are commonly linked, but this hypothesis has not yet been tested.

In particular, knowledge is lacking on how food neophobia and other factors jointly contribute to the rejection of insects as food and the relative weight of these factors. For this reason, in this paper, we analyze if some of these factors and food neophobia are related and if they jointly contribute to the rejection of insects as food, as previously mentioned by other authors [15,45,46] and, in particular, regarding disgust and food neophobia.

In order to better explain insect phobia, we hypothesize that the neophobia scale needs to be accompanied by a customized scale for insects.

In this study, through 420 questionnaires, we introduce a food neophobia scale and compare the results to those obtained using a neophobia scale. We measure the marginal effects that the two scales have on the behavior of respondents by measuring the intent and likelihood of eating insects in the near future.

## 2. Materials and Methods 

### 2.1. Data Collection and Sample

Data were collected in Pisa (Italy) in September 2019. To capture the maximal span of variation, the data were collected on different days, at different times, and in different buildings across the university and city during cultural events in which university venues were only the physical locations in which the questionnaire was conducted. A total of 420 respondents were used for the analysis. As already done by other studies (see for example Sogari et al. [23], or Boncinelli et al. [47]), our respondents are from a single location. Although there are different preferences for traditional cuisine based on different regions of Italy, we can reasonably assume that these preferences do not correlate or interact with intention or probability to eat insect. This assumption is based on the evidence that insects are novel foods for all Italians.

Without mentioning the objective of the research, the respondents were asked if they were willing to participate in a questionnaire. Indeed, their participation was voluntary, and they were not paid. We recruited even 23 vegetarian and vegan respondents that we excluded from the analysis considering that the object of study is proteins of animal origin. The evidence that a group of vegetarians and vegans was willing to participate to the survey is not surprising since we did not mention the object of the research. Moreover, we carried out a hypothetical study therefore we did not invite participants to eat any foods. The data were collected using a self-reported questionnaire. The sample consisted of 223 females (53% of total sample) and 197 males (47% of total).

The age of the participants ranged from 19 to 35 years; the average age of the sample was 23 years and the median age was 23. This particular statistic was linked to the university environment, and therefore the level of education was very high (undergraduate or graduate degree). Descriptive statistics of the sample are presented in Table 1.

### 2.2. Questionnaire Design

The structure of the questionnaire was based on previous study designs [16,23,48]. The introduction section of the questionnaire included demographic questions about the respondents, while the other sections were organized as described below.

The first section included 10 items concerning a person’s rejection or avoidance of unfamiliar food (food neophobia). Faccio et al. [49] argued that “food neophobia, seems to be an extremely complex attitude” and can vary during the course of one’s life [50]. Why a person is neophobic and what factors tend to maintain the neophobia over time are not well known. Many studies have reported that food neophobia is a major barrier to the acceptance of and readiness to try novel foods [51]. In particular, food neophobia can be analyzed using a specific scale called the Food Neophobia Scale (FNS) [29]. The FNS consists of five neophilic and five neophobic statements about food or situations related to food consumption (Table 2). A seven-point agreement scale, ranging from “1 = strongly disagree” to “7 = strongly agree” is used for responses.

The second section included 6 items that focused on some factors that could limit the consumption of insects (disgust, negative taste and texture, fear, low level of food safety, unsuitability, and social acceptance). According to previous works [16,48,52], these items were considered to be part of a single scale, named the Insect Phobia Scale (IPS) (Table 3). Inside the acceptance/rejection of insect-based foods, the first three items were codified as social factors and the other three items were codified as personal factors.

Similar to the FNS, a seven-point agreement scale was used for responses.

The last section explored two possible attitudes of respondents, i.e., the probability of eating food containing insects in the coming months and the intention to eat food containing insects in the coming months. In this case, a seven-point agreement scale, ranging from “1 = strongly disagree” to “7 = strongly agree” was also used.

In order to create the FNS and IPS, a reliability coefficient (Cronbach’s alpha) was used to check if the variables were related to each other and to describe the phobia. This coefficient measures reliability or internal consistency and it is useful to describe latent variables, and is often used to understand if multiple-question Likert scale surveys are reliable [53].

### 2.3. Statistical Analyses

Two logit models were implemented as follows: In Model 1, the dependent variable is the probability of eating food containing insects in the coming months. This variable is 1 if the probability of eating food containing insects is high (responses more than 4), and 0 otherwise. Therefore, *p_if_* is the probability that the *i*-th consumer will eat food containing insects, and this behavior can be modelled as follows:

(1) $$p_{if}=\alpha \;+\;\beta_{1}sex\;+\;\beta_{2}neophobia\;+\;\beta_{3}insect\;phobia\;+\;\varepsilon_{i},$$

where sex is equal to 1 if male or 0 if female, neophobia is the value obtained by the sum of responses about FNS, and insect phobia is the value obtained by the sum of responses about IPS. The value *α* is a constant term and *βs* measure the causal effect of covariates of the probability of eating food containing insects. Finally, *ε_i_* is the error term.

We estimated Model 2 with the same specification as Equation (1). In this case, the dependent variable was the intention to eat food containing insects in the coming months. Given the nature of our respondents, we excluded other individual sociodemographic characteristics as the low variance in the panels, such as age or education. 

## 3. Results

First, we verified that the questions related to the neophobia scales were related to each other and could describe phobia for new foods (Table 4 shows statistical data for the questions). In this regard, the questions were analyzed using Cronbach’s alpha and revealed a value of 0.83. Given the positive result, the neophobia variable was generated by adding the scores obtained for each respondent related to the food neophobia scale, taking into account that some authors [54,55] consider Likert scales with five or more categories to be continuous variables. The values of this variable range from 10 (low) to 60 (high level of neophobia).

The same procedure was followed for questions in the Insect Phobia Scale (Table 5 shows the statistical data for questions related to the IPS). The cross-correlations among the answers to the questions were analyzed using Cronbach’s alpha, revealing a value of 0.83. In this case, the result showed the congruence of the answers to describe insect phobia. The variable insect phobia was generated by adding the scores obtained for each respondent related to the Insect Phobia Scale. The values of this variable range from 10 (low) to 40 (high level of insect phobia). 

The results of the first logit regression model (Model 1) are shown in Table 6. The likelihood ratio test compares the null logit model with our model and shows that Chi-squared (3) = 59.00 Prob > Chi-squared = 0.000. Therefore, there is evidence of the effects of our covariates on the likelihood of eating food that contains insects. Moreover, pseudo-R^2^ equals 0.10, revealing that our variables explain a part of the total variance.

Gender is not a statistically significantly variable. Therefore, males and females have the same probability of eating food with insects. The statistical significance and the negative sign of coefficients show that a high probability of eating food containing insects is related to both those who have a low level of phobia for new foods and those who have a low level of phobia for eating insects. Indeed, the odds of eating food with insects is 4% lower if the level of neophobia measured by the neophobia scale increases one point and it is 7% lower if the insect phobia increases one point. The statistical significance, the positive sign and the large magnitude of constant term indicates that a female with no neophobia and no insect phobia is very likely to eat food with insect (i.e., 15 times higher).

The results of the second logit regression model (Model 2) are shown in Table 7. The likelihood ratio test shows that Chi-squared (3) = 96.00 Prob > Chi-squared = 0.000. Therefore, even for Model 2, there is evidence of the effects of our covariates on the intention to eat food containing insects. In this case, pseudo-R^2^ almost doubles, with a value of 0.18, i.e., our variables explain a substantial part of the total variance.

The results in terms of statistical significance, magnitude, and the sign of the coefficient are the same as the previous model. The results show that a high intention to eat food containing insects is related to both those who have a low level of phobia for new foods and those who have a low level of phobia for eating insects. An increase of 10 points in the neophobia scale reduces the intention to eat food containing insects by almost 50%. The same change in the insect phobia scale reduces the intention to eat food that contains insects twofold.

**Figure 1:**
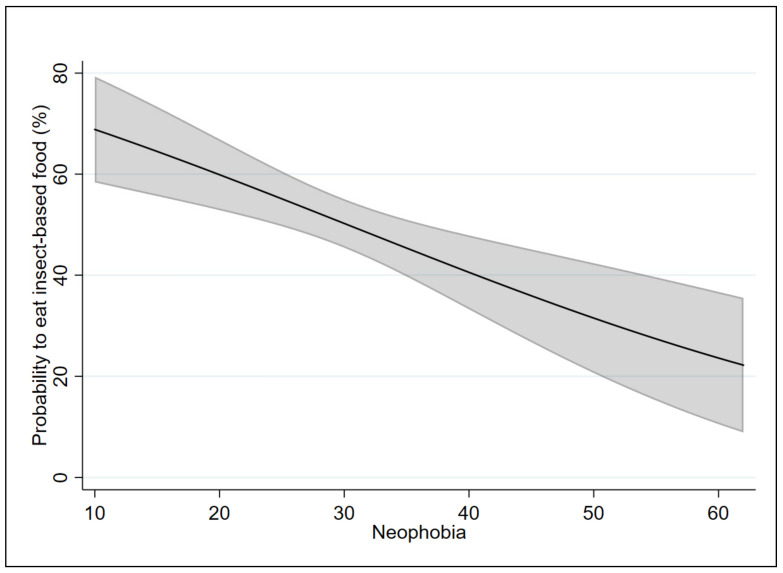
Marginal effects of the neophobia scale on Model 1 with 95% confidence intervals.

**Figure 2:**
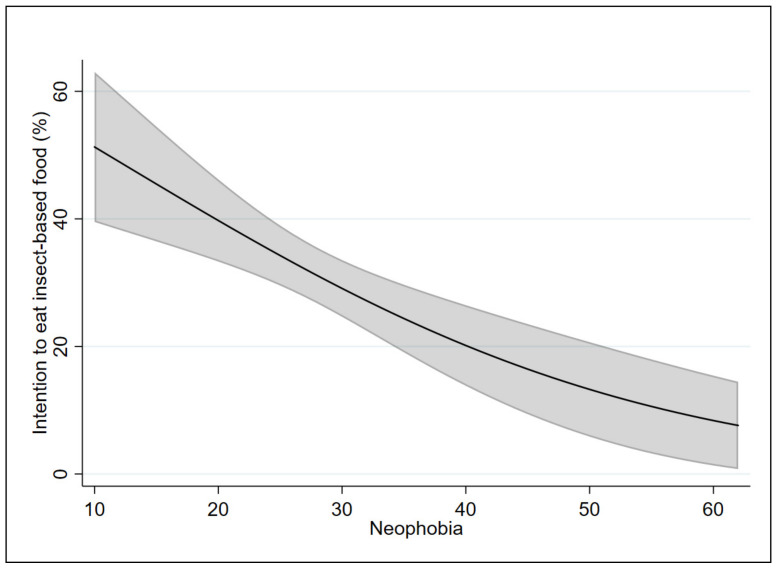
Marginal effects of the neophobia scale on Model 2 with 95% confidence intervals.

**Figure 3:**
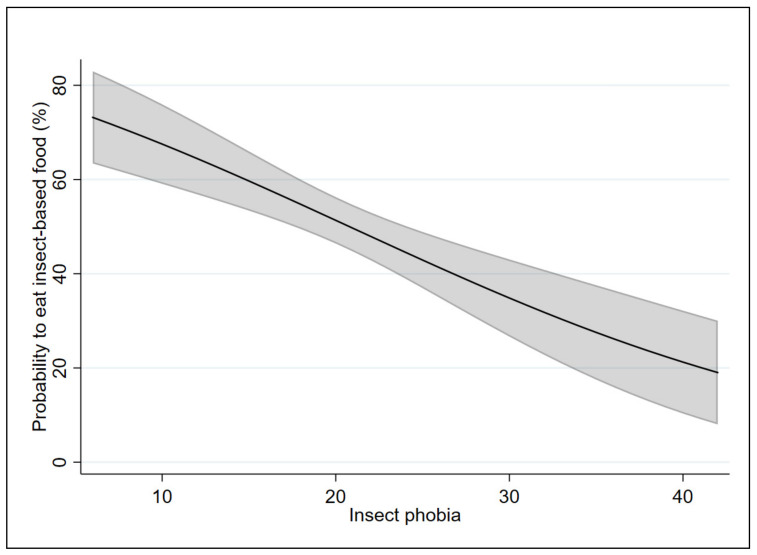
Marginal effects of the insect phobia scale on Model 1 with 95% confidence intervals.

**Figure 4:**
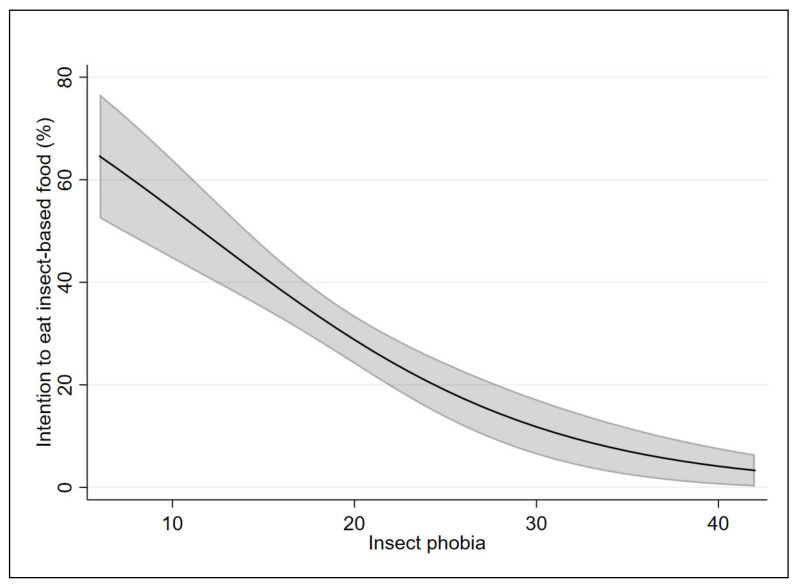
Marginal effects of the insect phobia scale on Model 2 with 95% confidence intervals.

In order to test our hypothesis (i.e., that the two scales affect the probability and the intention to eat food containing insects differently), the marginal effects of the applied models were analyzed. Figure 1 and Figure 2 show the marginal effects of the FNS on Models 1 and 2, respectively. Figure 3 and Figure 4 show the marginal effects of the IPS on Models 1 and 2, respectively. The marginal effects were calculated at different levels of neophobia (from 10 (low level) to 60 (high level) of neophobia) and at different levels of insect phobia (from 10 (low level) to 40 (high level) of insect phobia).

Considering Model 1, at a low level of neophobia (10), a person has a 69% probability of eating food containing insects in the coming months, while, at a high level of neophobia (60), this probability is equal to 24%.

Considering Model 2, at a low level of neophobia (10), the respondents have a 51% probability of intending to eat food containing insects in the coming months as compared with 8% probability for a person with a high level of neophobia (60).

Considering Model 1, a respondent with a low level of insect phobia (10) has a 67% likelihood of eating food containing insects in the coming months as compared with 21% likelihood for a respondent with a high level of insect phobia (40).

Considering Model 2, at a low level of insect phobia (10), there is a 54% probability that a respondent is willing to eat food containing insects in the coming months, while at a high level of insect phobia (40), the probability is equal to 4%.

By comparing the results of the two models, the probability of eating food containing insects decreases from 69% (for those who have a low level of neophobia) to 67% (for those who have a low level of insect phobia). The intention to eat food containing insects increases from 51% (for those who have a low level of neophobia) to 54% (for those who have a low level of insect phobia).

The probability of eating food containing insects decreases from 24% (for those have a high level of neophobia) to 21% (for those who have a high level of insect phobia). Similarly, the intention to eat food containing insects decreases from 8% (for those have a high level of neophobia) to 4% (for those who have a high level of insect phobia). 

## 4. Discussion

This study outlines some interesting findings, some of which also align with the data available in literature on this topic. First, we observed a relationship between neophobic consumers and the probability of eating food containing insects [20,22,30,31,32,37,42,56]. In particular, neophobic consumers are far less accepting of entomophagy than neophilic consumers [34,57].

Our findings confirmed the results that some authors found for specific factors, which, in a separate way, also contribute to the rejection of insects as food. The psychological factors are among the main barriers to acceptance [58,59]. In addition, the disgust factor is the most common reason for refusing an insect-based product [23,52,60,61]. The food exposure factor has a positive effect by increasing the familiarity of the product and also influences the acceptance of a new food, by decreasing neophobic reactions [62,63].

In contrast with some previous studies [16,32,52,64], gender did not have a significant influence on the neophobic level. We tested and confirmed the initial hypothesis that the two scales analyzed (FNS and IFS) give different results. On the basis of the fact that the neophobia scale has an effect on the willingness to eat insects, it should be considered to be a generic scale, used for all dishes outside the culinary tradition of the respondent.

In this regard, a specific scale for insects was tested and the effect it had on the probability and intention to eat insects was subsequently analyzed. There is a correlation between the two scales, but they can measure different effects. Notably, the IFS has an increasing impact on the intention to eat food containing insects as compared with neophobia. Instead, considering the probability of eating food containing insects, the FNS is slightly higher than the IFS. These differences confirm that the NFS is not sufficient to explain consumer behavior. Despite these results, the present study has some limitations. Our study only focused on a single country and city; therefore, the results should be replicated in different cultural contexts. As suggest by La Barbera et al. [46], “such cross-cultural validation could start with additional Western cultures but should eventually extend to non-Western cultures where entomophagy is traditionally more acceptable”. In addition, the focus on a relatively small study sample of young people “implies that the findings of this study cannot be readily generalized to other parts of society where the eating of insects is uncommon. Nevertheless, our insights show the most relevant determinants that are probably (at least partly) transferable to other study populations. Further studies in other countries are therefore recommended” [30]. Further validation should include more tests of cross-cultural measurements. However, the questionnaire was not directed towards specific targets and the survey itself was not based on any explicit selection mechanism. On the contrary, the literature developed in Italy includes papers based on severely self-selected samples. Some authors recruited respondents only in a single Italian city [65], in its main shopping mall [43], or from a university or its immediate surroundings [1,20,23,48,58,66].

It should be highlighted that, although most studies have been based on non-representative samples, the outcomes are widely consistent across the literature. This suggests that the main drivers related to the choice of consuming (or not) insect-based food are similar across populations, and their geographical locations. Although the literature has reported few differences related to the measurement of the “effect” of some specific variables, our results are essentially in line with those relative to other Western countries.

Considering that, in the future, insect phobia will have more of an effect than food neophobia on the intention to eat food containing insects, increasing familiarity will not be enough for consumers to adopt insect-based food, because the positive effect of increased familiarity could be thwarted by the disgust generated from a negative experience about consumption. This aspect could be overcome by investing in advertising messages with gastronomic and sensory characteristics.

Indeed, future research should include sensorial analyses of products containing insects in order to analyze consumers’ preferences. For this purpose, during interviews, the use of images or the tasting of real insect-based foods should be seriously considered. These techniques would help to describe a more realistic scenario of the consumption of insect-based food and could gather more accurate information on consumers’ behaviors.

Considering our country specifically, one possible strategy for improving the acceptance of insect-based foods could be to “hide”, i.e., incorporate, insects in foods, mainly in the well-known ones (e.g., pasta, pizza, and bread).

## 5. Conclusions

Although the idea of insect-based foods has been gaining acceptance over the years, consumers of Western countries continue to have many prejudices, which contribute to making it impossible (currently) to introduce foods containing insects into a normal diet because people consider this food to be as useless as it is disgusting. With this in mind, our results could be important for those involved in the entomophagy sector. For example, from the point of view of managers, a better understanding of consumer behavior toward these products could help them create specific marketing practices. At the same time, this could increase the demand for insect-based food products and, consequently, could enhance the income of food industries.

## Figures and Tables

**Table 1:** Characteristics of the sample.

Variable		Statistics
Respondents (no.)		420
Gender (%)	Male	47
	Female	53
Age (median/average)		23
Previous insect consumption (%)	yes	10
	no	90

**Table 2:** The Food Neophobia Scale (FNS).

Number	Statement
1	I am constantly sampling new and different foods.
2	I don’t trust new foods.
3	If I don’t know what a food is, I won’t try it.
4	I like foods from different cultures.
5	Ethnic food looks too weird to eat.
6	At dinner parties, I will try new foods.
7	I am afraid to eat things I have never had before.
8	I am very particular about the foods I eat.
9	I will eat almost anything.
10	I like to try new ethnic restaurants.

**Table 3:** Insect Phobia Scale (IPS).

Number	Statement
1	The idea of eating insects causes me disgust/repulsion.
2	Insect consumption is not socially acceptable.
3	I’m afraid insect-based foods have an unpleasant taste.
4	I’m afraid insect-based foods have an unpleasant consistency.
5	I think insect-based foods have poor hygiene.
6	I think that eating insects is not suitable for our diet.

**Table 4:** The Food Neophobia Scale (FNS).

Number	Statement	Median	IQR
1	I am constantly sampling new and different foods (R).	3	2
2	I don’t trust new foods.	3	2
3	If I don’t know what a food is, I won’t try it.	4	3
4	I like foods from different cultures (R).	2	3
5	Ethnic food looks too weird to eat.	2	2
6	At dinner parties, I will try new foods (R).	2	2
7	I am afraid to eat things I have never had before.	3	3
8	I am very particular about the foods I eat.	4	3
9	I will eat almost anything (R).	2	2
10	I like to try new ethnic restaurants (R).	2	3

R, reverse coded and IQR, interquartile range.

**Table 5:** Insect Phobia Scale (IPS).

Number	Statement	Median	IQR
1	The idea of eating insects causes me disgust/repulsion.	4	3
2	Insect consumption is not socially acceptable.	2	3
3	I’m afraid insect-based foods have an unpleasant taste.	4	3
4	I’m afraid insect-based foods have an unpleasant consistency.	4	3
5	I think insect-based foods have poor hygiene.	3	2.5
6	I think that eating insects is not suitable for our diet.	2	3

IQR = interquartile range.

**Table 6:** Results of the first logit model (Model 1) of the probability to eat food that contains insects.

Variables	Odds Ratio	Standard Errors	z	*p* > |z|
Sex	1.03	0.22	0.14	0.886
Neophobia	0.96	0.01	−3.51	0.000
Insect phobia	0.93	0.02	−4.39	0.000
Constant	14.90	6.23	6.46	0.000

**Table 7:** Results of the second logit model (Model 2) of the intention to eat food that contains insects.

Variables	Odds Ratio	Standard Errors	z	*p* > |z|
Sex	1.34	0.32	1.23	0.217
Neophobia	0.95	0.01	−3.90	0.000
Insect phobia	0.89	0.02	−5.76	0.000
Constant	17.12	8.16	5.95	0.000
